# Sphingolipid metabolism is associated with osteosarcoma metastasis and prognosis: Evidence from interaction analysis

**DOI:** 10.3389/fendo.2022.983606

**Published:** 2022-08-29

**Authors:** Xinyue Hu, Xin Zhou, Jue Zhang, Liangliang Li

**Affiliations:** ^1^ School of Medicine, Southeast University, Nanjing, Jiangsu, China; ^2^ Department of Orthopaedic surgery, The Affiliated Jiangning Hospital of Nanjing Medical University, Nanjing, Jiangsu, China

**Keywords:** sphingolipid metabolism, osteosarcoma, metastasis, prognosis, model

## Abstract

**Background:**

Metabolism is widely involved in the occurrence and development of cancer. However, its role in osteosarcoma (OS) has not been elucidated.

**Methods:**

The open-accessed data included in this study were downloaded from The Cancer Genome Atlas (TCGA) database (TARGET-OS project). All the analysis was performed in R environments.

**Results:**

Based on the single sample gene set enrichment analysis algorithm, we quantified 21 metabolism terms in OS patients. Among these, sphingolipid metabolism was upregulated in the metastatic OS tissue and associated with a worse prognosis, therefore aroused our interest and selected for further analysis. Our result showed that sphingolipid metabolism could activate the Notch signaling and angiogenesis pathway, which might be responsible for the metastasis ability and poor prognosis. A protein-protein interaction network was constructed to illustrate the interaction of the differentially expressed genes between high and low sphingolipid metabolism. Immune analysis showed that multiple immune terms were upregulated in patients with high sphingolipid metabolism activity. Then, a prognosis model was established based on the identified DEGs between patients with high and low sphingolipid metabolism, which showed great prediction efficiency. Pathway enrichment showed the pathway of myogenesis, spermatogenesis, peroxisome, KRAS signaling, pancreas beta cells, apical surface, MYC target, WNT beta-catenin signaling, late estrogen response and apical junction was significantly enriched in high risk patients. Moreover, we found that the model genes MAGEB1, NPIPA2, PLA2G4B and MAGEA3 could effectively indicate sphingolipid metabolism and risk group.

**Conclusions:**

In summary, our result showed that sphingolipid metabolism is associated with osteosarcoma metastasis and prognosis, which has the potential to be a therapeutic target for OS.

## Introduction

Osteosarcoma (OS) is one of the most prevalent primary bone malignancy all over the world, especially in children and adolescents (1). Due to the characteristics of high invasiveness, OS patients tend to have a poor prognosis (2). Surgery combined with chemotherapy is the first-line therapy option for OS. While advancement has been made in the OS treatment, disease distant metastasis can dramatically impair the prognoses of patients (3). Therefore, the identification of novel biomarkers indicating the diagnosis and treatment of OS is important.

Tumor metabolism is vital to meet the metabolic demands associated with tumor proliferation and expansion of cancer cells (4). Widespread metabolic reprogramming in the tumor microenvironment can maintain cancer cell survival under the pressure of nutritional stress (5). Recently, researchers are increasingly interested in the crosstalk between metabolic changes and cancer development. Wang et al. found that metabolic reprogramming of T lymphocyte activation regulated by the transcription factor Myc is the main driving force for proliferating cells to utilize glutamine (6). Wu et al. revealed that under anoxia conditions, OMA1/OPA1 axis was activated and could increase mitochondrial reactive oxygen species to stabilize HIF-1α, further promoting glycolysis and progression of colon cancer (7). Meanwhile, cell metabolism is controlled by multi-level regulation (8). Studies have reported that the metabolizing fuel produced by autophagy can provide metabolic plasticity for cancers, which promotes the growth and survival of cancer cells (9). Moreover, targeting tumor metabolism might be a promising therapy option for cancers (10). In OS, Lv et al. found that β-Phenethyl Isothiocyanate could induce cell death of OS by altering iron metabolism, disturbing the redox balance, and activating the MAPK signaling (11). Hu et al. indicated that fructose-coated angstrom silver could suppress OS growth and metastasis by facilitating ROS-dependent apoptosis through the alteration of glucose metabolism (12).

In our study, we found that the sphingolipid metabolism level was upregulated in metastatic OS patients and associated with a poor prognosis. Moreover, Notch signaling and angiogenesis might be activated in patients with high sphingolipid metabolism activity. Meanwhile, we found that sphingolipid metabolism was associated with multiple immune terms, indicating that sphingolipid metabolism might affect tumor biological processes through disturbing cancer immune status. Finally, a prognosis model based on MAGEB1, NPIPA2, PLA2G4B and MAGEA3 was established, which had a good prognosis prediction efficiency. Pathway enrichment analysis was then performed to explore the underlying biological differences between high and low risk groups. Furthermore, we found that MAGEB1, NPIPA2, PLA2G4B and MAGEA3 could indicate sphingolipid metabolism level and risk group.

## Methods

### Data source

The open-accessed expression profile and clinical information were downloaded from the TCGA database (https://portal.gdc.cancer.gov/, TARGET-OS project). The expression profile was originally STAR-counts form and then collated to TPM form using R code. Human Genome reference file GRCh38 obtained from the Ensembl website was used for original probe annotation. All the data were preprocessed before the analysis, including probe annotation, data consolidation, and batch normalization.

### Single sample gene set enrichment analysis

Clusterprofiler and GSVA package in R environment was used to perform ssGSEA analysis, which can calculate enrichment score based on the expression profile (13). The reference pathway information was shown in **Table S1**. The immune-related terms were obtained from a previous study (14).

### Differentially expressed genes analysis

For the selected two groups, DEGs analysis was performed using the limma package with the threshold of |logFC| > 1 and P < 0.05 (15).

### Protein-protein interaction network

PPI network was established based on the STRING database (https://cn.string-db.org/). In detail, the “Organism” was “Homo”. The nodes with high confidence (0.900) were identified. Cytoscape v3.7.2 was used for network visualization. Cytoscape software was used for network visualization. ClueGO plug-in in cytoscape was used to perform biological enrichment analysis for the nodes (16).

### Prognosis model construction and evaluation

For the selected genes, univariate Cox regression analysis was firstly performed to identify the prognosis-related genes. Furthermore, LASSO regression analysis was used for dimensionality reduction. Multivariate Cox regression analysis was finally performed for prognosis model construction. All the patients would be assigned a riskscore with the formula of “ Riskscore = Coef A * Gene A + Coef B + Gene B + … + Coef N * Gene B” (17). According to median riskscore, patients were divided into high and low risk groups. Kaplan-Meier survival curve was performed to evaluate the survival difference between high and low risk groups. Receiver operating characteristic (ROC) curve.

### Pathway enrichment analysis

Pathway enrichment analysis between high and low risk groups was performed using the GSEA algorithm (18). The reference pathway file was Hallmark, c2.cp.kegg.v7.5.1.symbols and c5.go.v7.5.1.symbols gene set.

### Statistical analysis

Statistical analysis was performed using the R software v4.0. In all cases, P.values under 0.05 were considered statistically significant. Students T-tests were used for the continuous variables normally distributed while Mann-Whitney U tests were used for the non-normally distributed continuous variables.

## Results

### Metabolism terms identification

**Figure 1:**
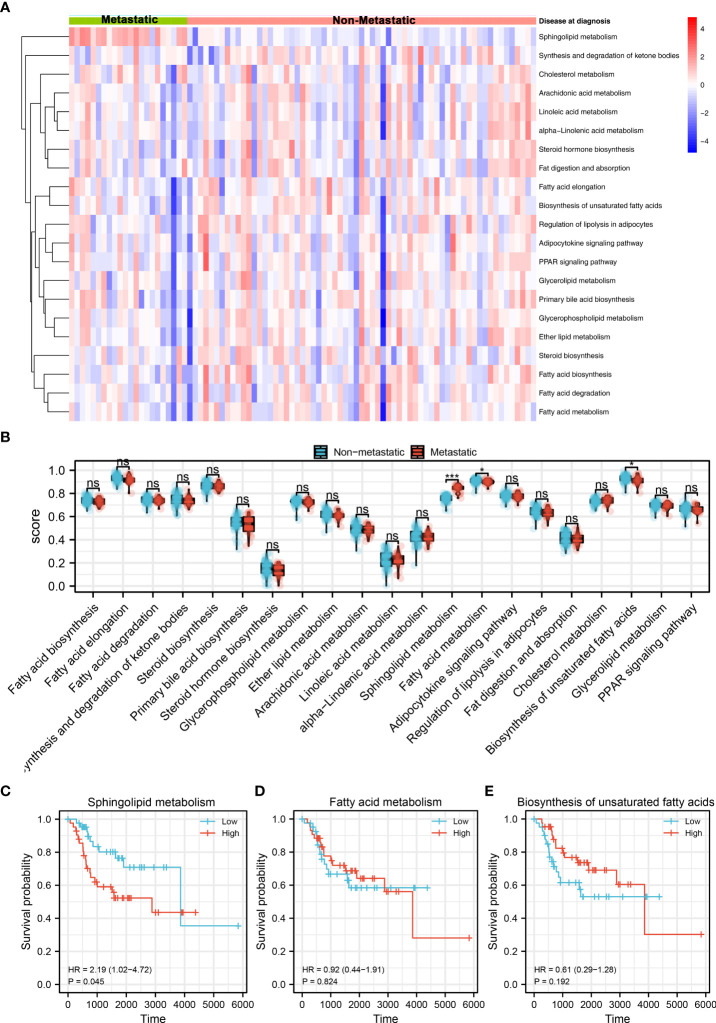
Identification of sphingolipid metabolism in OS Notes: **(A)**: A total of 21 metabolism terms were quantified using the ssGSEA algorithm, which was shown in a heatmap; **(B)**: The pathway activity of 21 metabolism terms in metastatic and non- metastatic OS, in which fatty acid metabolism and biosynthesis of unsaturated fatty acids were downregulated, while the sphingolipid metabolism was upregulated in the metastatic OS; **(C)**: Kaplan-Meier survival curve of overall survival between high and low sphingolipid metabolism patients; **(D)**: Kaplan-Meier survival curve of overall survival between high and low fatty acid metabolism patients; **(E)**: Kaplan-Meier survival curve of overall survival between high and low biosynthesis of unsaturated fatty acids patients. ns, P < 0.05. *P < 0.05; ***P < 0.001.

The brief flowchart of our study was shown in **Figure S1**. Based on the ssGSEA algorithm, 21 metabolism terms were quantified, which was shown in **Figure 1A**. Then, we evaluated the difference of these terms between metastatic and non-metastatic OS. The result showed that fatty acid metabolism and biosynthesis of unsaturated fatty acids were downregulated, while the sphingolipid metabolism was upregulated in the metastatic OS (**Figure 1B**). Further, we performed Kaplan-Meier survival curves analysis of these three terms. The result showed that sphingolipid metabolism might be associated with a poor prognosis of OS patients (**Figure 1C**, HR = 2.19, P = 0.045). However, no significant difference was observed in atty acid metabolism and biosynthesis of unsaturated fatty acids (**Figures 1D, E**). Therefore, the sphingolipid metabolism aroused our interest and was selected for further analysis.

### DEGs identification and PPI network

**Figure 2:**
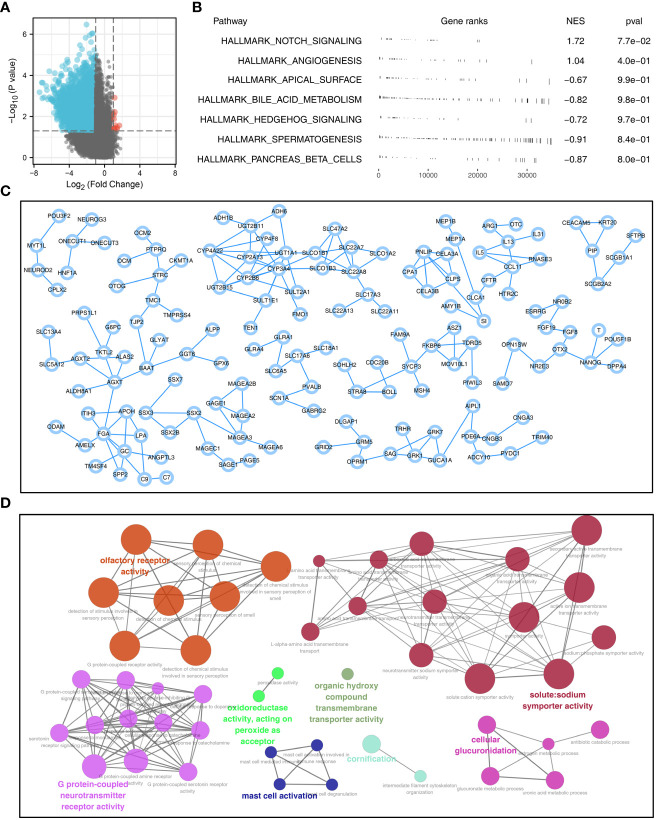
PPI network and pathway enrichment analysis of sphingolipid metabolism **(A)**: With the threshold of |logFC| > 1 and P < 0.05, 5969 downregulated and 11 upregulated genes were identified between patients with high and low sphingolipid metabolism activity; **(B)**: GSEA analysis was performed based on the Hallmark gene set to explore the biological differences between high and low sphingolipid metabolism patients; **(C)**: PPI network based on the DEGs was established to reveal potential protein interactions; **(D)**: ClueGO (a plug-in in Cytoscape software) analysis of the nodes.

With the threshold of |logFC| > 1 and P < 0.05, 5969 downregulated and 11 upregulated genes were identified between high and low sphingolipid metabolism patients (**Figure 2A**). GSEA analysis showed that the pathway of Notch signaling and angiogenesis were significantly enriched in high risk group (**Figure 2B**). A PPI network was constructed to illustrate the interaction between these DEGs (**Figure 2C**). ClueGO analysis showed that these DEGs were mainly enriched in the olfactory receptor activity, solute:sodium symporter activity, G protein-coupled neurotransmitter receptor activity, organic hydroxy compound transmembrane transporter activity, mast cell activation, cornification and cellular glucuronidation (**Figure 2D**).

### Sphingolipid metabolism was positively correlated with Notch signaling and angiogenesis

**Figure 3:**
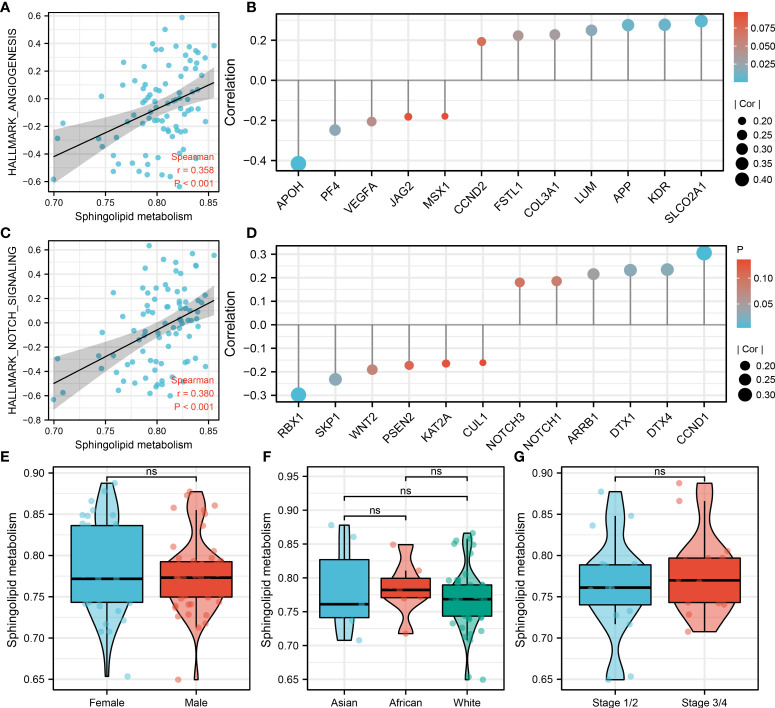
Sphingolipid metabolism was positively correlated with Notch signaling and angiogenesis Notes: **(A)**: Sphingolipid metabolism was positively correlated with angiogenesis; **(B)**: The association between the genes involved in angiogenesis and sphingolipid metabolism activity; **(C)**: Sphingolipid metabolism was positively correlated with Notch signaling; **(D)**: The association between the genes involved in the Notch signaling and sphingolipid metabolism activity; **(E)**: The sphingolipid metabolism difference in male and female patients; **(F)**: The sphingolipid metabolism difference in Asian, African and White patients; **(G)**: The sphingolipid metabolism difference in Stage 1/II and Stage III/IV patients. ns, P < 0.05.

Notch signaling and angiogenesis were reported to be associated with the distant metastasis of OS (19, 20). We found that sphingolipid metabolism was positively correlated with the Notch signaling and angiogenesis (**Figure 3A** and **Figure 3C**; Notch signaling, R = 0.358, P < 0.001; Angiogenesis, R = 0.380, P < 0.001). Among the genes involved in angiogenesis, sphingolipid metabolism was positively correlated with SLCO2A1, KDR, APP, LUM, COL3A1, FSTL1, CCND2, but negatively correlated with APOH, PF4, VEGFA, JAG2 and MSX1 (**Figure 3B**). Among the genes involved in Notch signaling, sphingolipid metabolism was positively correlated with CCND1, DTX4, DTX1, ARRB1, NOTCH1, NOTCH3, but negatively correlated with RBX1, SKP1, WNT2, PSEN2, KAT2A and CUL1 (**Figure 3D**). Next, we evaluated the sphingolipid metabolism between different populations, including gender, race and stage. However, no significant difference was observed (**Figure 3E, G**). Moreover, we try to explore the pathway activity difference of Notch signaling and angiogenesis in metastatic and non-metastatic OS (**Figure S2A**). The result showed that angiogenesis was remarkably upregulated in metastatic OS, yet no significant difference was observed in Notch signaling between metastatic and non-metastatic OS. For the genes involved in the Notch signaling and angiogenesis, only ARRB1 was found significantly upregulated in metastatic OS tissue (**Figures S2B, C**).

### Immune analysis

**Figure 4:**
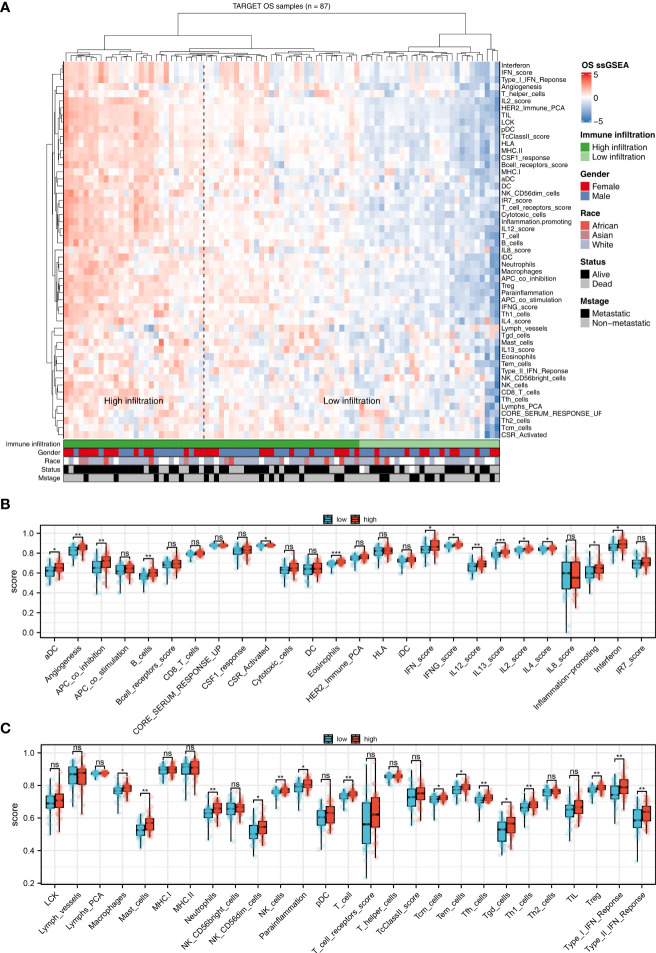
The correlation between sphingolipid metabolism and immune terms **(A)**: ssGSEA algorithm was performed to quantify the 53 immune terms; **(B, C)**: Immune terms difference between patients with high and low sphingolipid metabolism activity. ns, P < 0.05. *P < 0.05; **P < 0.01; ***P < 0.001.

Immune is closely related to metabolism (21). Therefore, we explored the underlying interaction between immune terms and sphingolipid metabolism. Based on the ssGSEA algorithm, 53 immune terms were quantified, which was shown in **Figure 4A**. The result showed that the terms of aDC, angiogenesis, APC_co_inhibition, B_cells, CSR_activated, eosinophils, IFN_score, IFNG_score, IL12 score, IL13 score, IL2 score, IL4 score, inflammation-promoting, interferon, macrophages, mast cells, neutrophils, NK_CD56dim_cells, NK_cells, parainflammation, T_cells, Tcm_cells, Tem_cells, Tfh_cells, Tgd_cells, Th1_cells, Treg, Type_I_IFN_Response and Type_II_IFN_Response were remarkably higher in high sphingolipid metabolism patients (**Figures 4B, C**).

### Prognosis model

**Figure 5:**
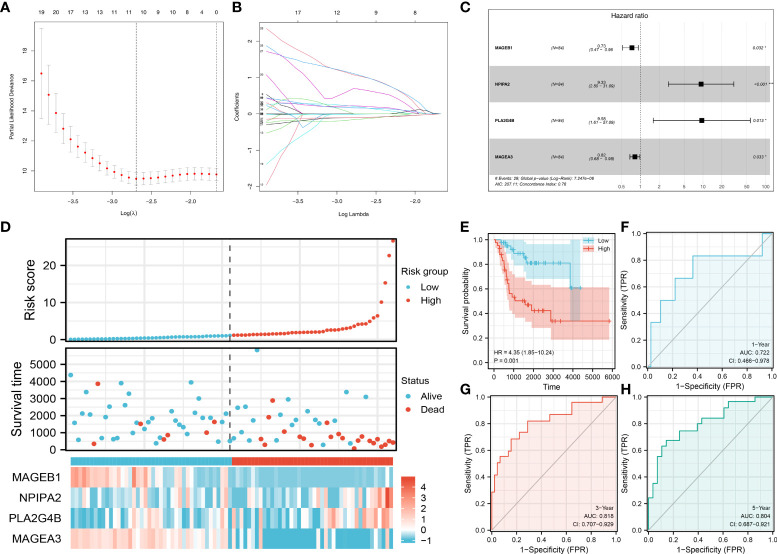
Prognosis model construction Notes: **(A, B)**: LASSO regression analysis was used for dimensionality reduction; **(C)**: Multivariate Cox regression analysis identified four genes for model construction, including MAGEB1, NPIPA2, PLA2G4B and MAGEA3; **(D)**: The overview of the prognosis model; **(E)** Kaplan-Meier survival curve of overall survival between high and low risk patients; **(F–H)**: ROC curves of 1-, 3-, 5-years overall survival.

Based on the DEGs identified between high and low sphingolipid metabolism patients, we try to establish a prognosis indicating the prognosis of OS patients. Univariate Cox regression analysis was firstly performed to identify the prognosis-related genes. Then, LASSO regression analysis was used for dimensionality reduction (**Figures 5A, B**). Multivariate Cox regression analysis identified four genes for model construction, including MAGEB1, NPIPA2, PLA2G4B and MAGEA3 (**Figure 5C**). A prognosis model was constructed with the formula of “Riskscore = MAGEB1 * -0.309 + NPIPA2 * 2.233 + PLA2G4B * 2.259 + MAGEA3 * -0.200”. A higher percentage of dead cases was observed in high risk patients (**Figure 5D**). Kaplan-Meier survival curve showed that the patients in high risk group might have a worse prognosis compared with that in low risk group (**Figure 5E**). ROC curves showed that our model had a great prediction efficiency in 1-, 3, and 5- years survival (**Figures 5F–H**, 1-year AUC = 0.722, 3-year AUC = 0.818, 5-year AUC = 0.804). Also, we evaluated the prediction efficacy of our model in patients disease-free survival (**Figure S3**). Kaplan-Meier survival curve showed that the patients in high risk group might have a worse disease-free survival (**Figure S3A**). ROC curves indicated a good prediction efficiency in pateints 1-, 3- and 5-year disease-free survival (**Figure S3B**).

### Pathway enrichment analysis

**Figure 6:**
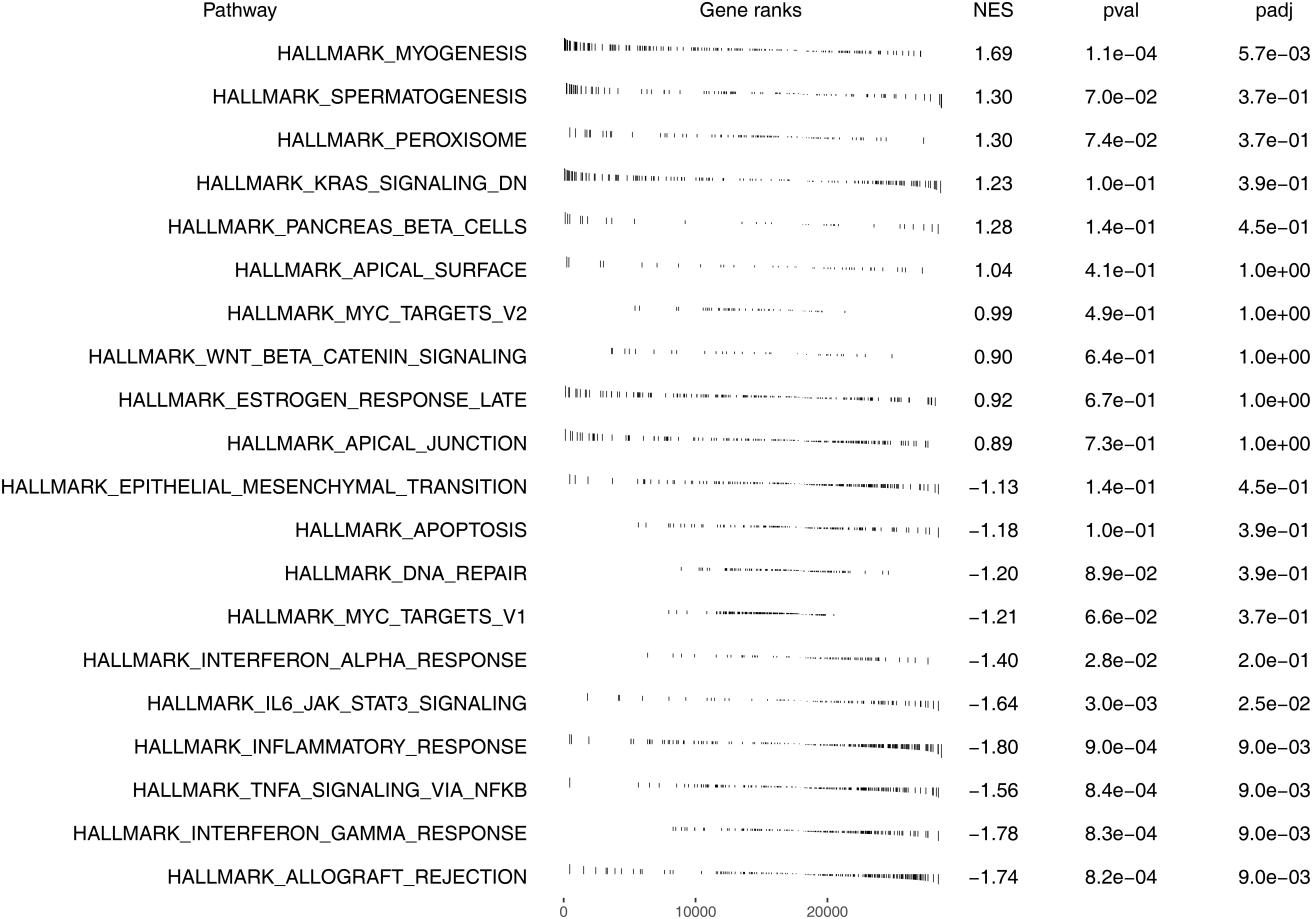
Pathway enrichment analysis of our model based on Hallmark gene set.

We next explored the underlying biological differences between high and low risk patients. The result showed that the pathway of myogenesis, spermatogenesis, peroxisome, KRAS signaling, pancreas beta cells, apical surface, MYC target, WNT beta catenin signaling, late estrogen response and apical junction were significantly enriched in high risk patients (**Figure 6**). Kyoto Encyclopedia of Genes and Genomes (KEGG) analysis showed that the terms of type II diabetes mellitus, cardiac muscle contraction, dilated cardiomyopathy, hypertrophic cardiomtopathy hcm, amyotrophic lateral sclerosis als were significantly enriched in high risk group (**Figure S4A**). Gene oncology (GO) analysis showed that the terms of regulation of transmembrane transport, actin binding, muscle system process, muscle organ development, muscle contraction were significantly enriched in high risk group (**Figure S4B**).

### MAGEB1, NPIPA2, PLA2G4B and MAGEA3 could indicate sphingolipid metabolism and risk group

**Figure 7:**
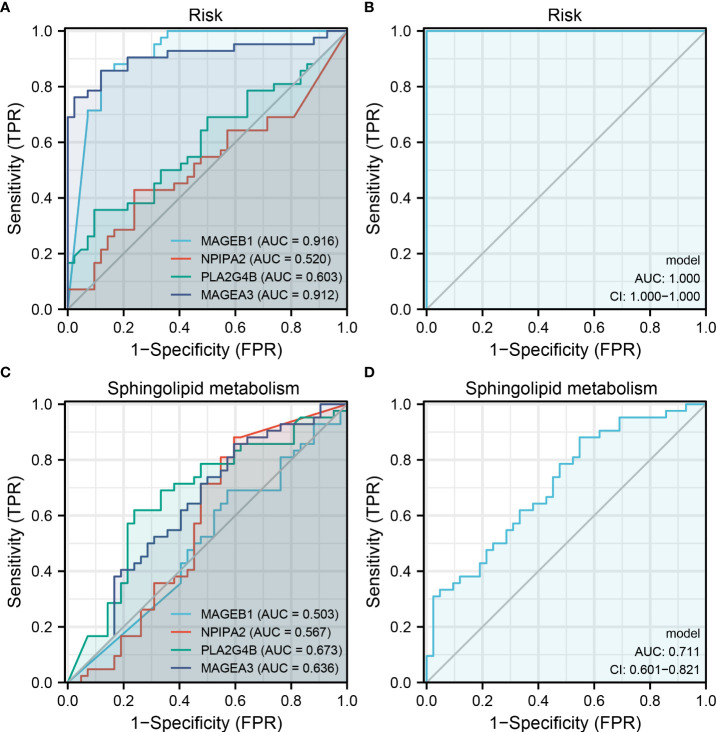
MAGEB1, NPIPA2, PLA2G4B and MAGEA3 could indicate sphingolipid metabolism and risk group Notes: **(A)** ROC curve of MAGEB1, NPIPA2, PLA2G4B and MAGEA3 on risk group prediction; **(B)** The logistic regression model of these four genes on risk group prediction (17.8664 + -155.4921*MAGEB1 + 894.5988*NPIPA2 + 1238.153*PLA2G4B + -87.4001*MAGEA3); **(C)** ROC curve of MAGEB1, NPIPA2, PLA2G4B and MAGEA3 on sphingolipid metabolism group prediction; **(D)** The logistic regression model of these four genes on sphingolipid metabolism group prediction (1.2038 + 0.2264*MAGEB1 + 0.4005*NPIPA2 + -3.024*PLA2G4B + -0.2842*MAGEA3).

Our prognosis model identified four genes, including MAGEB1, NPIPA2, PLA2G4B and MAGEA3. We next evaluated the prediction efficiency of these four model genes on risk group and sphingolipid metabolism level. The result showed that these four genes had an extremely great prediction efficiency in patients risk group (**Figure 7A**, MAGEB1, AUC = 0.916, NPIPA2, AUC = 0.520, PLA2G4B, AUC = 0.603, MAGEA3, AUC = 0.912). After logistic regression, the AUC value of these four genes could be increased to 1.0 (**Figure 7B**). As for the sphingolipid metabolism level, the AUC value of MAGEB1, NPIPA2, PLA2G4B and MAGEA3 were 0.503, 0.567, 0.673 and 0.636, respectively (**Figure 7C**). After logistic regression, the AUC value of these four genes could be increased to 0.711 (**Figure 7D**).

## Discussion

OS is still a serious public health problem that results in an approximately 2.4% death rate in child cancers all over the world (22). Lung is the most common site of OS metastasis and lung metastases often result in a poor prognosis (1).

Recently, tumor metabolism has gradually attracted attention in cancer research (23). Cells must increase their intake of nutrients from the environment to meet their biosynthetic needs related to proliferation. However, compared with normal cells, the proliferation of tumor cells is often accompanied by extensive metabolic reprogramming (24). Metabolite signaling is involved in the regulation of malignant transformation, cell proliferation, epithelial-mesenchymal transformation, differentiation block and cancer dryness, as well as inflammatory response and immune surveillance in the tumor microenvironment (24).

To the best of our knowledge, this is the first study that comprehensively explored the metabolism in OS based on bioinformatic analysis. In our study, we firstly quantified 21 metabolism terms based on the ssGSEA algorithm. Among these, sphingolipid metabolism was upregulated in the metastatic OS tissue and associated with a worse prognosis, therefore aroused our interest and selected for further analysis. Our result showed that sphingolipid metabolism could activate the Notch signaling and angiogenesis pathway, which might be responsible for the metastasis ability and poor prognosis. Immune analysis showed that multiple immune terms were upregulated in patients with high sphingolipid metabolism level. Then, a prognosis model was established based on the identified DEGs between patients with high and low sphingolipid metabolism, which showed great prediction efficiency. Pathway enrichment showed that the pathway of myogenesis, spermatogenesis, peroxisome, KRAS signaling, pancreas beta cells, apical surface, MYC target, WNT beta catenin signaling, late estrogen response and apical junction were significantly enriched in high risk patients. Moreover, we found that the model genes MAGEB1, NPIPA2, PLA2G4B and MAGEA3 could effectively indicate sphingolipid metabolism and risk group. In the clinical setting, it can help to predict the metabolic status and grouping of patients by detecting the expression of these four genes in cancer tissue, which would contribute to the prognosis prediction and treatment choice.

Sphingolipid metabolism has been found wide involved in cancer occurrence and development of cancer (25). Ceramides is one of the principal components of sphingolipid. Elsherbini et al. indicated that the ceramides are important for the exosome formation, which might be an underlying target of cancer therapy (26). Moreover, as an intercellular signaling molecule that plays a role in embryogenesis, sphingosine‐1‐phosphate (S1P) is also involved in the formation of new blood vessels during embryonic development, which would also be utilized by cancer cells. Researchers have found that S1P is the key regulator of angiogenesis in multiple cancers based on mice models (27, 28). Metastasis requires cells to undergo structural and signaling changes. As an important component of the plasma membrane, gangliosides strongly regulate cell adhesion/movement, leading to tumor metastasis (29). Cortini et al. found that S1P might be a valuable therapeutic target for OS patients by exploring the metabolic adaptations to the acidic microenvironment of OS cells (30). Our result showed that the patients with a higher sphingolipid metabolism level tend to experience distant metastasis, which might be an underlying metabolism target of OS therapy options.

Notch signaling is involved in many aspects of cancer biology (31). In OS, Qin et al. found that Notch signaling could regulate OS proliferation and migration through Erk phosphorylation based on *in vivo* and *in vitro* experiments (32). Also, Dai et al. indicated that the inhibition of the Notch signaling pathway attenuates the progression of OS cell motility, metastasis, and epithelial-mesenchymal transition (33). Angiogenesis is important for tumor cancer cell survival and metastasis (34). Jian et al. found that TSP-1 could promote OS angiogenesis and metastasis through CD36/vasculostatin signaling axis (20). Li et al. found that exosomal lncRNA OIP5-AS1 could regulate OS tumor angiogenesis and autophagy through miR-153 and ATG5 (35). Our result showed that the Notch signaling and angiogenesis pathways were positively correlated with sphingolipid metabolism. The immune microenvironment is tightly associated with cell metabolism. Our result showed that sphingolipid metabolism was associated with multiple immune terms, including neutrophils, Treg, macrophages, et al., indicating that sphingolipid metabolism might affect tumor biological processes through disturbing cancer immune status. For example, Charan et al. found that tumor secreted ANGPTL2 could facilitate the recruitment of neutrophils to the lung, further promoting lung pre-metastatic niche formation of OS (36).

Some limitations should be noticed. Firstly, the population enrolled in this study was mainly the white population. Meanwhile, the race of a considerable part of the population is unknown, which might lead to underlying race bias. Secondly, the sample counts with complete expression profiles and clinical information are small (less than 100), which might reduce the credibility of the article to some extent.

## Data availability statement

The original contributions presented in the study are included in the article/**Supplementary Material**. Further inquiries can be directed to the corresponding author.

## Author contributions

XH and LL were responsible for the conception, design. XH and XZ were responsible for the development of methodology and analysis. XH and JZ performed the bioinformatics analysis. XH, XZ, JZ and LL wrote and revised the draft. LL final approval of the submitted version. All authors read and approved the final manuscript.

## Conflict of interest

The authors declare that the research was conducted in the absence of any commercial or financial relationships that could be construed as a potential conflict of interest.

## Publisher’s note

All claims expressed in this article are solely those of the authors and do not necessarily represent those of their affiliated organizations, or those of the publisher, the editors and the reviewers. Any product that may be evaluated in this article, or claim that may be made by its manufacturer, is not guaranteed or endorsed by the publisher.

## Supplementary material

Supplementary Figure 1 The flowchart of the whole study.

Supplementary Figure 2 Notch signaling and angiogenesis in metastatic and non-metastatic OS **(A)**: The pathway activity difference of Notch signaling and angiogenesis in metastatic and non-metastatic OS; **(B)**: Angiogenesis-related genes in metastatic and non-metastatic OS; **(C)**: Notch signaling-related genes in metastatic and non-metastatic OS.

Supplementary Figure 3 Disease-free survival difference of our model **(A)**: Kaplan-Meier survival curve of disease-free survival; **(B)**: ROC curves of 1-, 3- and 5-years disease-free survival.

Supplementary Figure 4 GO and KEGG analysis of our model **(A)**: KEGG analysis of our model; **(B)**: GO analysis of our model.
