# Supplementary figures and images for: Sphingolipid metabolism is associated with osteosarcoma metastasis and prognosis: Evidence from interaction analysis
Source: Front Endocrinol (Lausanne). 2022 Aug 29;13:983606. doi: 10.3389/fendo.2022.983606 (PMC9465041; doi:10.3389/fendo.2022.983606)

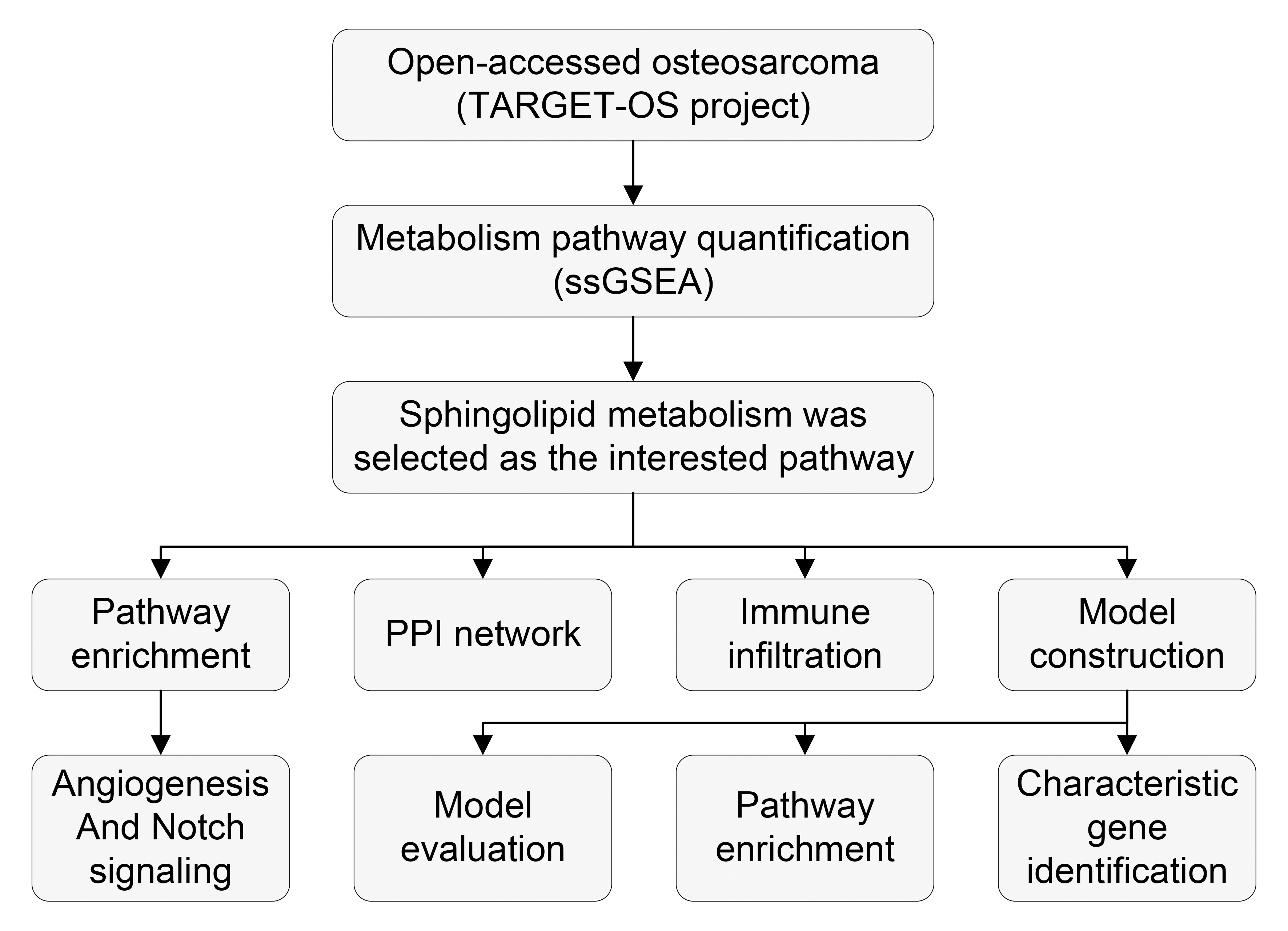

Supplement: Supplementary Figure 1 — The flowchart of the whole study. [file Image_1.tif]

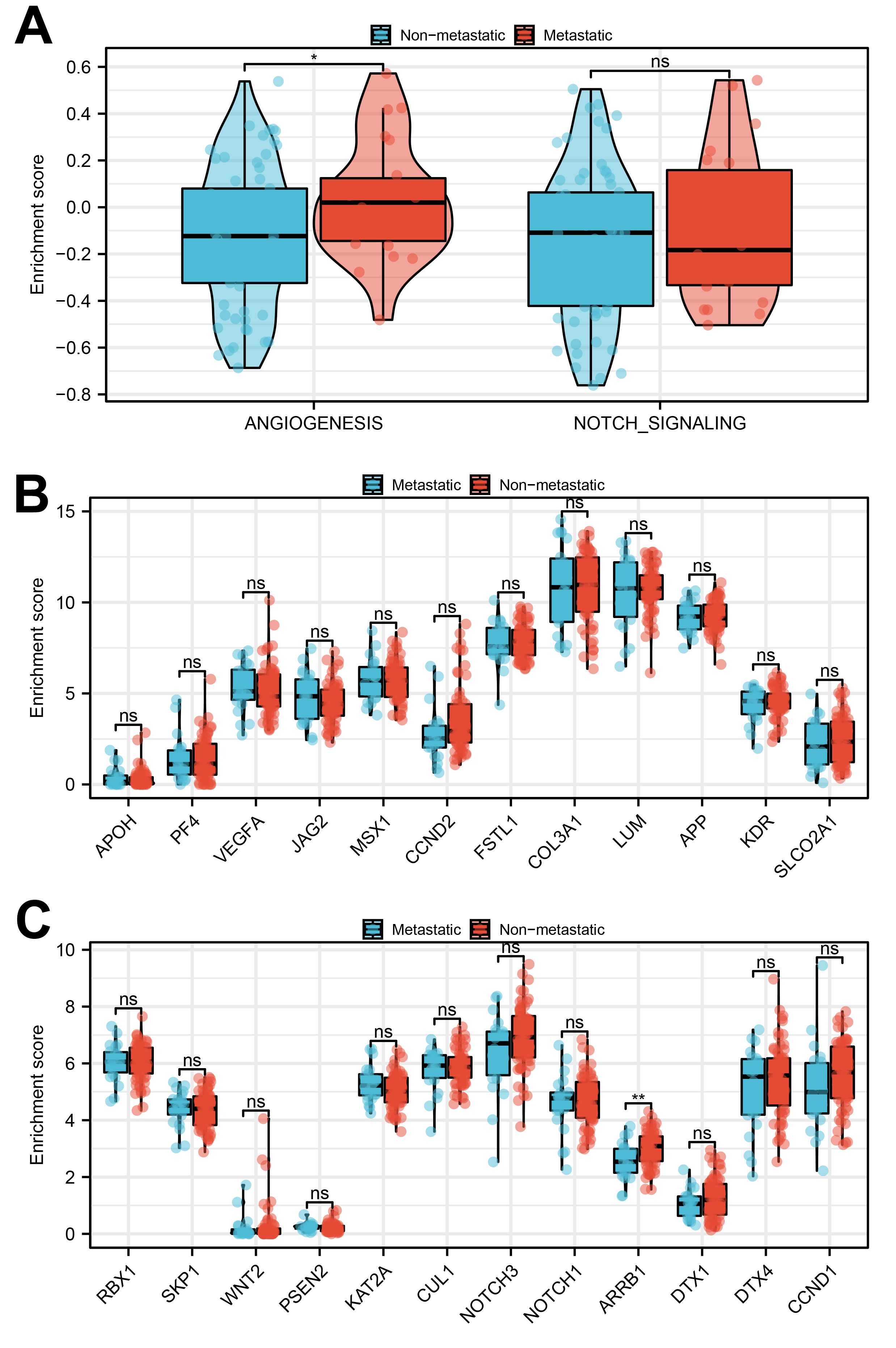

Supplement: Supplementary Figure 2 — Notch signaling and angiogenesis in metastatic and non-metastatic OS (A): The pathway activity difference of Notch signaling and angiogenesis in metastatic and non-metastatic OS; (B): Angiogenesis-related genes in metastatic and non-metastatic OS; (C): Notch signaling-related genes in metastatic and non-metastatic OS. [file Image_2.tif]

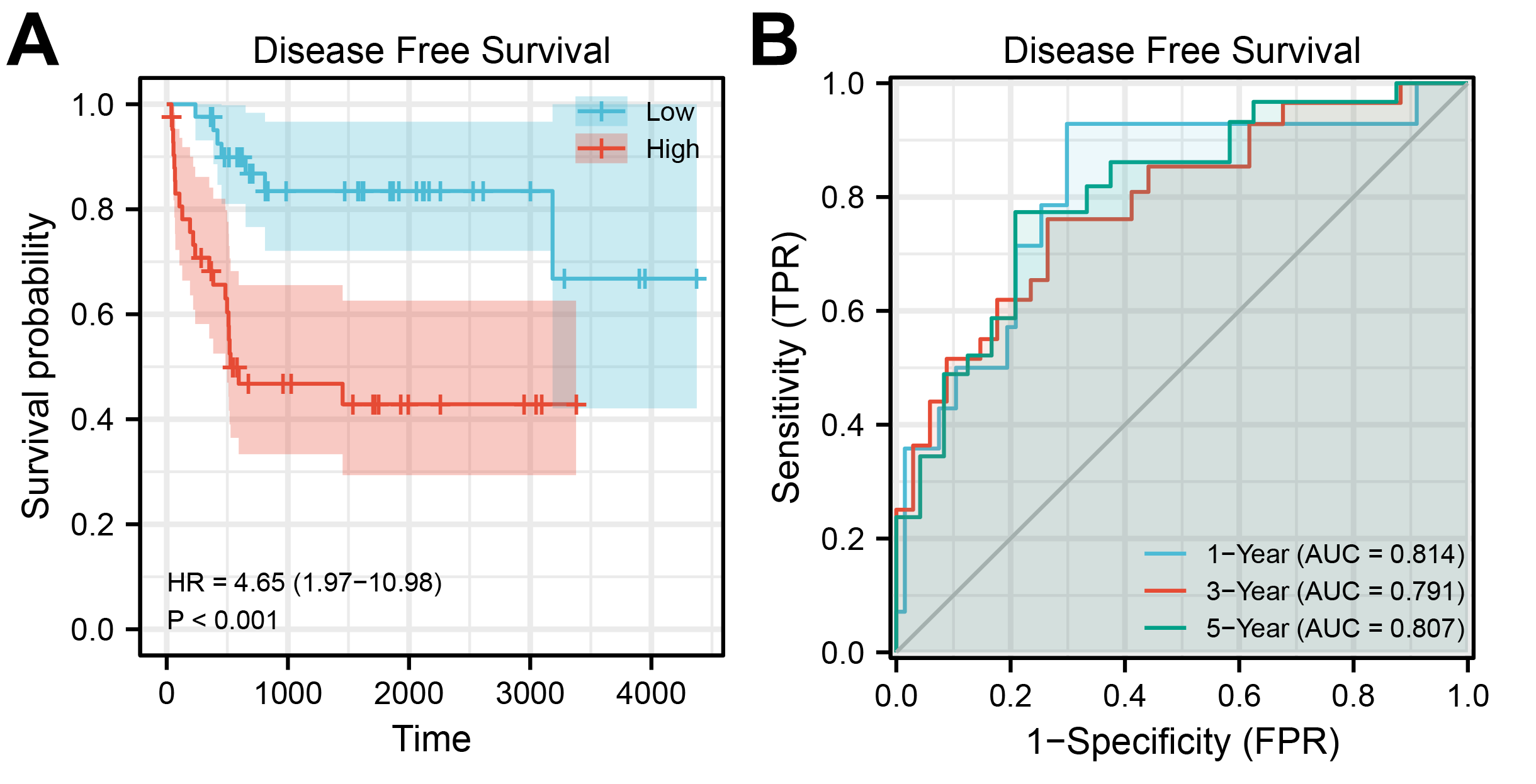

Supplement: Supplementary Figure 3 — Disease-free survival difference of our model (A): Kaplan-Meier survival curve of disease-free survival; (B): ROC curves of 1-, 3- and 5-years disease-free survival. [file Image_3.tif]

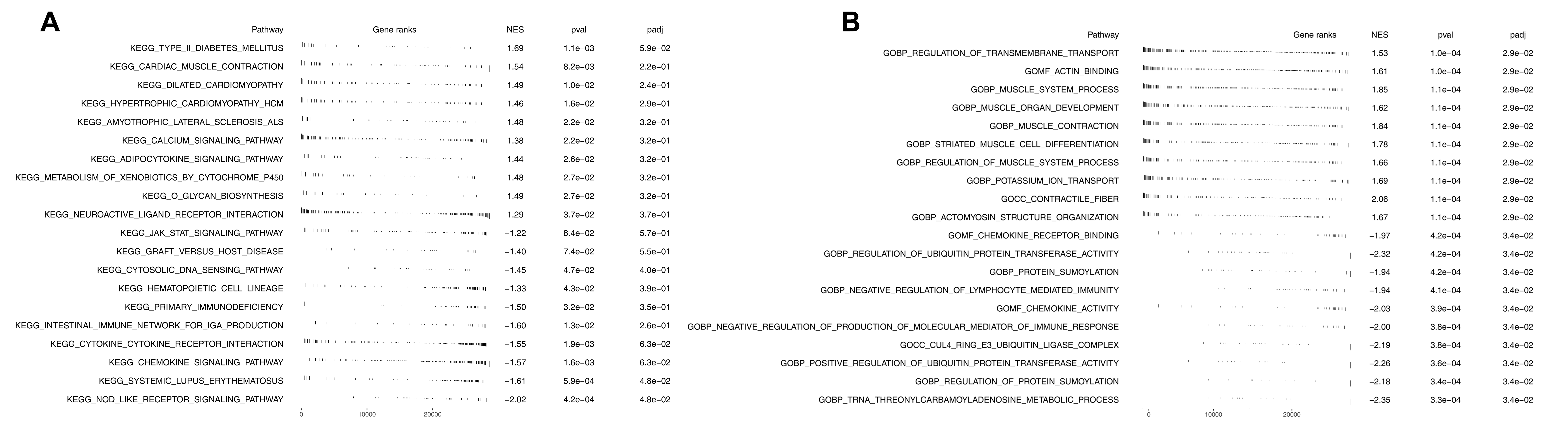

Supplement: Supplementary Figure 4 — GO and KEGG analysis of our model (A): KEGG analysis of our model; (B): GO analysis of our model. [file Image_4.tif]
